# Sick building syndrome: do outdoor pollutants and pollen affect it?

**DOI:** 10.3389/falgy.2024.1383079

**Published:** 2024-07-05

**Authors:** Sandra Nora González-Díaz, Grecia Jaqueline Hernández-Salcido, Cindy Elizabeth de Lira-Quezada, Jorge Alberto Cantú-Hernández, Carlos Macouzet-Sánchez, Alejandra Macias-Weinmann, Natalhie Acuña-Ortega

**Affiliations:** ^1^ Faculty of Medicine, Autonomous University of Nuevo León, Monterrey, Mexico; ^2^ Dr. José Eleuterio González, University Hospital, Monterrey, Mexico; ^3^ Regional Center of Allergy and Clinical Immunology, Monterrey, Mexico

**Keywords:** nasal symptoms, outdoor pollutants, pollen, rhinitis, sick building syndrome (SBS)

## Abstract

**Introduction:**

Sick building syndrome (SBS) refers to non-specific complaints, including upper-respiratory irritative symptoms, headaches, fatigue, and rash, which are usually associated with a particular building by their temporal pattern of occurrence and clustering among inhabitants or colleagues. The aim of the study was to determine the association between the clinical manifestations of sick building syndrome with outdoor pollutants and airborne pollen.

**Methods:**

It was a descriptive and prospective observational study conducted from November 2021 to April 2022. It included subjects over 18 years old who completed an online survey on sick building syndrome (general symptoms, nasal, ocular, oropharyngeal, and skin symptoms) presented at home, housing information and personal history. The APS-330 from Pollen Sense ® was used to obtain data on pollen in the air and the local pollution monitoring system (SIMA) to obtain information regarding pollutants. For statistical analysis, SPSS version 16 was used.

**Results:**

A total of 402 surveys were included; 91% of the subjects reported having at least 1 symptom. Females presented more general symptoms (fatigue and headache) than males. Subjects with a personal history of atopy showed a higher prevalence of practically all symptoms. Airborne pollen exposure was positively associated with mucosal symptoms in eyes and nose. Outdoor fungi spore exposure was positively associated with oculo-nasal and cutaneous symptoms in the scalp.

**Conclusion:**

This study found significant associations with female gender and a history of atopy, which suggests a higher risk for these subjects. Despite the limitations of the study, we can conclude that there is an association between the clinical manifestations of sick building syndrome with indoor and outdoor pollution.

## Introduction

Sick building syndrome (SBS) refers to non-specific complaints, including upper-respiratory irritative symptoms, headaches, fatigue, and rash, which are usually associated with a particular building by their temporal pattern of occurrence and clustering among inhabitants or colleagues (1).

This definition first appeared in 1970 when researchers noticed a high prevalence of symptoms linked to inhabiting buildings that relied on mechanical ventilation systems for the movement of fresh air, temperature regulation, and humidity control. They also noted that the increased use of synthetic materials in building construction, the rise in the number of workers employed in office settings, and the automation of office work with its associated increased regulation and stress all played a role. Buildings are not the only places where this phenomenon can happen; home environments must be considered too (2). Understanding indoor air quality, which is defined as the total human exposure to chemical pollutants in the air, is crucial to comprehending this phenomenon. It is well known that there is an overlap between indoor and outdoor pollution, especially particulate matter (PM) and volatile organic compounds (COV) (3, 4). The development of this illness has been linked to various personal variables, including female sex, a history of allergy diseases, and smoking (5).

Recent research has focused on indoor pollution and sick building syndrome, particularly in office settings, only a few researches have examined the SBS in relation to the home environment but given that most individuals now spend their time at home, this information is crucial. Most studies only evaluate indoor pollution, rarely considering outdoor pollutants or climate variations. However, the accelerated urbanization and economic development have resulted in an increase in environmental degradation that is getting worse and more pervasive. Extreme weather has also been brought on by global warming, which has led to health issues for the general population (6). Increased morbidity may result from the short- and long-term impacts of high levels of air pollution on human health (4). Outdoor pollution can result from chemicals or contaminants of biological origin modified by climate change or human activity, such as bioaerosols and aeroallergens (7). These particle pollutants, some of which may include those from vehicle exhaust and some combustion products, can enter buildings through windows or vents and pollute the air inside of them (8).

Another factor that can increase indoor and outdoor pollution and exacerbate allergic respiratory illnesses is allergens. Pet skin flakes, rodent urine, molds, or arthropod feces (such as those from house dust mites and cockroaches) can all be sources of indoor allergic pollutants. Aeroallergens from weeds, trees, grasses, or molds are examples of outdoor allergies. Additionally, interior aeroallergen concentration is influenced by outside pollen. Complex bioaerosol dynamics control the number of allergens in the interior environment (7). It is fascinating to observe how these elements have a detrimental effect on people's health and influence environmental or external pollution.

WHO implemented SARS-CoV-2 health policies in nations all over the world due to the pandemic crisis. Following the WHO's “stay home” mantra, isolation was one of the most crucial steps in breaking the virus's chain of transmission. Due to the amount of time people spend inside their homes, this policy may have an impact on both interior and exterior pollution and create personal and environmental risks. As a result, people may unintentionally be exposed to the factors that lead to sick building syndrome's clinical manifestations (9). The aim of the study was to determine the association between the clinical manifestations of sick building syndrome with outdoor pollutants and pollen levels in the air.

## Materials and methods

A descriptive, cross-sectional study was carried out in subjects over 18 years old living in Monterrey and the metropolitan area, individuals who did not meet these criteria were excluded. The participants in this study were volunteers who gave their consent to fill out the pre-designed survey through the “Google Forms” platform, and that was distributed electronically (Supplementary Material S1). The survey is a version in Spanish obtained from Mentese, S., & Tasdibi, D. (2016). Before requesting consent, the participants were explained in detail about the aims and benefits of this study.

The investigation was conducted by the department of Allergy and Clinical Immunology of the University Hospital “Dr. José Eleuterio González” in Monterrey, Nuevo León, Mexico. The study took place from November 2021 to April 2022 and was approved by the ethics committee of this institution (approval number AL21-00015).

### Assessment of sick building syndrome (SBS) symptoms

For the evaluation of the symptoms of SBS, the questions were divided into 3 groups: general symptoms (fatigue and headache), mucosal symptoms (itching, burning or irritation of the eyes, irritation, congestion or runny nose and hoarse throat and skin) and skin symptoms (red or dry facial skin, flaking/itchy scalp, dry hands, itchy or red skin). The participants answered if these symptoms appeared frequently (daily), sometimes (weekly) or never in the last month.

### Assessment of personal factors

For the evaluation of personal factors, we included questions about gender, smoking habit and personal history of atopy diagnosed by a doctor (rhinitis/conjunctivitis/atopic dermatitis and asthma). History of atopy and active smoking were considered as confounding variables.

### Assessment of building characteristics

Questions about the physical characteristics of the dwelling were included: age of the building, size of the house in accordance with the Housing Building Code in Mexico, 2010, floor number in which the dwelling lives (horizontality/verticality of the dwelling), number of people living in the dwelling, number of rooms.

### Assessment of exposure to outdoor air pollutants, meteorological factors and airborne pollen levels

The data for the evaluation of outdoor pollution was acquired from the Environmental Monitoring System (SIMA) of Nuevo León, including the following meteorological parameters (temperature, humidity and wind speed), and air quality evaluators (particulate matter less than 10 micrometers (PM10), particulate matter less than 2.5 micrometers (PM2.5), ozone (O3), sulfur dioxide (SO2), nitrogen dioxide (NO2) and carbon monoxide (CO), which are considered the main particles pollutants in Monterrey and the metropolitan area, according to SIMA through the 13 stations that operate in 11 of the 12 municipalities that make up the metropolitan area of Monterrey. Measurements were made using the following methods: carbon monoxide (CO) with infrared photometry; ozone (O3) with UV spectrophotometry; for nitrogen dioxide (NO2) gas phase chemiluminescence is used; sulfur dioxide (SO2) with pulsating UV fluorescence; particles smaller than 10 micrometers (PM10) Beta ray attenuation; particles smaller than 2.5 micrometers (PM2.5) with Beta ray attenuation and white light scattering. The data obtained in the SIMA monitoring network equipment is extracted from each of the stations to carry out an automatic validation process, this allows it to be compared with the requirements established by the Official Mexican Standards.

We included the address of each participant to assign them to each of the SIMA environmental monitoring stations. The geolocation subjectś homes and the stations was obtained by converting geographic coordinates (latitude and longitude) to UTM projection coordinates. A monthly average of the outdoor pollutants was made at the time of answering the survey and then compared with the symptom information.

To evaluate the association between the airborne pollen levels and the presence of the clinical manifestations of SBS, the APS-330 from the Pollen Sense system was used, which is a real time air particle sensor and is located 12 meters above the ground, on the roof of an out-patient and consults building in the city of Monterrey, Nuevo Leon, Mexico.

The following pollens were included: *Quercus spp, Fraxinus spp, Populus spp, Cupressaceae spp, Gramineaes spp, Chenopodium spp/amaranthus spp, Alternaria spp, Cladosporium spp, Aspergillus spp, Penicillium spp*, biological debris, and other particles (organic and inorganic particles smaller than 5 μm). The data was downloaded monthly through the “PollenWise” application.

### Statistical analysis

Chi-square test was used for the assessment of differences in demographic and indoor features between genders, and in the comparison of SBS symptoms prevalence personal factors (gender, personal history of atopy and current smoking). Normal multiple logistic regression models were used to assess the relationship between SBS symptoms (general, mucosal, and cutaneous) with exposure to outdoor air pollutants (nitrates, specifically NO, NO2 and NOx, and others like SO2, PM10, and PM2.5), meteorological parameters (temperature, relative humidity, atmospheric pressure, and wind speed), airborne pollen levels (from *Quercus spp* and *Fraxinus spp*) and fungi spores levels (*Aspergillus spp*), all adjusted for personal factors (gender, current smoking, and history of atopy) and some of them for meteorological parameters (Temperature and Wind Speed for air pollutants, and Relative Humidity for fungi spores levels). We based this decision on previous literature [Lu et al. (6)]. Pearson's correlation coefficient was obtained for the assessment of the correlation between air pollutants and meteorological parameters exposure. Results of the regression analysis were interpreted by odds ratio (OR) with 95% confidence interval (95% CI) where *p* value <0.05 was considered statistically significant. All statistical analyses were performed by SPSS software V26.0.

## Results

A total of 402 urban residents were recruited (Table 1). A total of 269(66.9%) were females, 140(35.3%) had a history of atopy (allergic asthma, allergic conjunctivitis or rhino-conjunctivitis, and atopic dermatitis), and 43(10.8%) were current smokers, without significant differences between genders. The mean age was higher for females than for males (31.87 ± 12.48 years vs. 29.33 ± 11.68 years, respectively; *p* = 0.05), and the mean number of inhabitants per living place was 3.63 ± 1.61.

**Table 1:** Demographic features from the patients and their living places.

Demographic features	General (*n* = 402)	Males (*n* = 133)	Females (*n* = 269)	*p*
Age (mean ± SD)	31.03 ± 12.26	29.33 ± 11.68	31.87 ± 12.48	0.050
Smoking *n* (%)	43 (10.8)	16 (12)	27 (10.2)	0.610
Personal history of atopy *n* (%)	140 (35.3)	39 (30)	101 (37.8)	0.146
Inhabitants per living place (mean ± SD)	3.63 ± 1.61	3.50 ± 1.39	3.70 ± 1.71	0.228
Years inhabiting the living place *n* (%)				
0–5 years	164 (40.8)	51 (38.3)	113 (42)	0.090
6–10 years	61 (15.2)	16 (12)	45 (16.7)	
11–20 years	80 (19.9)	25 (18.8)	55 (20.4)	
>20 years	97 (24.1)	41 (30.8)	56 (20.8)	
Living place's antiquity *n* (%)				
<10 years	120 (29.9)	28 (21.1)	92 (34.2)	0.008
>10 years	280 (69.7)	105 (78.9)	175 (65.1)	
Living place *n* (%)				
Departament (<75 m^2^)	97 (24.1)	30 (22.6)	67 (24.9)	0.623
House (>75 m^2^)	305 (75.9)	103 (77.4)	202 (75.1)	
Rooms per living place (mean ± SD)	7.15 ± 3.13	6.62 ± 3.11	7.42 ± 3.11	0.017
Floor *n* (%)				
First	128 (31.8)	43 (32.3)	85 (31.6)	0.097
Second	237 (59)	85 (63.9)	152 (56.5)	
Third	24 (6)	4 (3)	20 (7.4)	
Fourth	6 (1.5)	0 (0)	6 (2.2)	
≥Fifth	7 (1.7)	1 (0.8)	6 (2.2)	
Humidity issues *n* (%)				
Cristals’ condensation	109 (27.1)	39 (29.3)	70 (26)	0.596
Walls/roof damage	92 (22.9)	32 (24.1)	60 (22.3)	
Clothes/bed humidity	7 (1.7)	3 (2.3)	4 (1.5)	
Mold stains	39 (9.7)	8 (6)	31 (11.5)	
Mold smell	4 (1)	1 (0.8)	3 (1.1)	
None	151 (37.6)	50 (37.6)	101 (37.5)	
Window-opening routine *n* (%)				
Never	23 (5.7)	9 (6.8)	14 (5.2)	0.950
Sometimes	101 (25.1)	31 (23.3)	70 (26)	
Often	275 (68.4)	92 (69.2)	183 (68)	
Air conditioning system *n* (%)				
Air washer	52 (12.9)	14 (10.5)	38 (14.1)	0.261
Central air conditioner	6 (1.5)	4 (3)	2 (0.7)	
Mini-split	286 (71.1)	95 (71.4)	191 (71)	
None	58 (14.4)	20 (15)	38 (14.1)	
Time spent at home *n* (%)				
<12 h	158 (39.3)	73 (54.9)	85 (31.6)	<0.001
12–18 h	162 (40.3)	43 (32.3)	119 (44.2)	
19–24 h	82 (20.4)	17 (12.8)	65 (24.2)	
Time spent at work (hours) *n* (%)				
<12 h	234 (80.4)	89 (81.7)	145 (79.7)	0.609
12–18 h	56 (19.2)	20 (18.3)	36 (19.8)	
19–24 h	1 (0.3)	0 (0)	1 (0.5)	

Most of the residents had lived in their living place for more than ten years (69.7%), with a significant difference between males and females (78.9% vs. 65.1%, *p* = 0.008). Male residents also stayed significantly less in their living places than females (less than 12 h per day, 54.9% vs. 31.6% respectively; *p *≤ 0.001). When analyzing the proximity between the homes of the subjects considered for this study and the SIMA stations, it was found that the minimum distance was 1.98 m and the maximum distance was 11.16 km.

The prevalence of SBS symptoms (Table 2) was stratified by personal factors. Females presented more general symptoms (fatigue and headache) than males (216, 80.3% vs. 84, 63.2%; *p *≤ 0.001 and 188, 69.9% vs. 64, 48.1%; *p *≤ 0.001, respectively). Subjects with a personal history of atopy showed a higher prevalence of practically all symptoms, mainly in mucosal and cutaneous symptoms (*p *≤ 0.001 in most of the cases). Current smokers presented a significantly smaller prevalence in ocular (17, 38.6% vs. 213, 60%; *p* = 0.009) and oculo-nasal symptoms (12, 27.3% vs. 182, 51.3%; *p* = 0.004). The physical factors of the home were evaluated: age greater or less than 10 years and the size of the home greater or less than 75m2. We found a statistically significant association between the absence of nasal symptoms and living in a house larger than 75 m^2^ *p* 0.05 OR 0.88(95% CI 0.79–0.98) and spending less than 12 h at home *p* 0.037 OR 0.75(95% CI 0.59–0.96). There was no statistically significant association found in subjects’ symptoms who lived in homes over 10 years old.

**Table 2:** Prevalence of sick building symptoms stratified by demographic features.

Symptoms	Total (*n* = 402)	Gender			History of atopy			Current smoking		
		Males (*n* = 133)	Females (*n* = 269)	*p*	No	Yes	*p*	No	Yes	*p*
General symptoms										
Fatigue	300 (74.6)	84 (63.2)	216 (80.3)	<0.001	183 (70.9)	117 (81.8)	0.017	262 (73.8)	37 (84.1)	0.146
Headache	252 (62.7)	64 (48.1)	188 (69.9)	<0.001	154 (59.7)	98 (68.5)	0.085	226 (63.7)	24 (54.5)	0.251
Mucosal symptoms										
Ocular	232 (57.7)	77 (57.9)	155 (57.6)	1.000	117 (45.3)	115 (80.4)	<0.001	213 (60.0)	17 (38.6)	0.009
Nasal	282 (70.1)	86 (64.7)	196 (72.9)	0.105	158 (61.2)	123 (86.0)	<0.001	253 (71.3)	26 (59.1)	0.116
Oculo-nasal	196 (48.8)	63 (47.4)	133 (49.4)	0.751	89 (34.5)	107 (74.8)	<0.001	182 (51.3)	12 (27.3)	0.004
Nasopharyngeal	217 (54.0)	68 (51.1)	149 (55.4)	0.457	128 (49.6)	88 (61.5)	0.028	190 (53.5)	25 (56.8)	0.750
Cutaneous symptoms										
Face	224 (55.7)	60 (45.1)	164 (61.0)	0.003	123 (47.7)	100 (69.9)	<0.001	204 (57.5)	20 (45.5)	0.148
Scalp	176 (43.8)	54 (40.6)	122 (45.4)	0.394	89 (34.5)	86 (60.1)	<0.001	154 (43.4)	22 (50.0)	0.425
Hands	188 (46.8)	52 (39.1)	136 (50.6)	0.034	106 (41.1)	81 (56.6)	0.003	172 (48.5)	15 (34.1)	0.079

The physical factors of the home were evaluated: age greater or less than 10 years and the size of the home greater or less than 75 m^2^. We found a statistically significant association between the absence of nasal symptoms and living in a house larger than 75 m^2^ *p* 0.05 OR 0.88(95% CI 0.79–0.98) and spending less than 12 h at home *p* 0.037 OR 0.75(95% CI 0.59–0.96).

The exposure levels of outdoor air pollution and meteorological parameters (see Table 3) showed an average individual exposure (mean ± SD) to nitrates (NO, NO2 and NOx) of 13.16 ± 8.48 μg/m^3^, 17.64 ± 4.70 μg/m^3^ and 31.20 ± 12.13 μg/m^3^, respectively, and to SO2, PM10 and PM2.5 of 5.45 ± 1.38 μg/m^3^, 70.03 ± 12.05 μg/m^3^ and 23.15 ± 4.71 μg/m^3^, respectively. From these, only NOx and PM10 showed a mean level exposure over its safety limits (≥25 μg/m^3^ and ≥45 μg/m^3^, respectively) (9).

**Table 3:** Descriptive analysis of individual air pollution exposure levels and meteorological parameters.

	Mean ± SD	Minimum	25th percentile	50th percentile	75th percentile	Maximum
Air pollution exposure (ppb)						
NO	13.16 ± 8.48	4.76	7.41	10.88	15.53	62.57
NO2	17.64 ± 4.70	9.48	14.38	17.65	20.25	34.31
NOx	31.20 ± 12.13	14.51	22.62	27.40	36.80	85.06
SO2	5.45 ± 1.38	2.53	4.46	5.60	6.51	8.50
PM10	70.03 ± 12.05	46.81	60.65	67.98	83.42	97.91
PM2.5	23.15 ± 4.71	12.25	20.80	22.13	25.65	39.02
Meteorological parameters exposure						
Temperature (°C)	20.18 ± 3.52	13.6	18.30	20.50	22.20	26.1
Relative humidity (%)	50.62 ± 12.11	23.69	40.45	51.58	59.26	75.74
Pressure (atm)	715.11 ± 6.34	696.71	711.29	714.68	719.33	751.37
Wind speed (km/h)	9.03 ± 2.51	3.00	7.16	9.49	10.21	22.53

Correlation between outdoor air pollution and meteorological parameters was calculated (see Table 4). The weak correlation between meteorological factors (mainly temperature, humidity and wind speed) permitted to adjust for these factors when investigating associations between air pollutants and SBS symptoms.

**Table 4:** Correlation between calculated individual levels of air pollutants and meteorological parameters.

Value	NO	NO2	NOx	SO2	PM10	PM2.5	Temperature	Relative humidity	Wind speed
NO	1	0.561***	0.919***	0.204***	0.003	0.288***	−0.431***	0.100*	−0.153**
NO2		1	0.793***	0.176***	0.179***	0.395***	−0.711***	0.105*	−0.360***
NOx			1	0.205***	0.010	0.334***	−0.628***	0.159**	−0.263***
SO2				1	0.318***	0.348***	−0.111*	−0.611***	0.003
PM10					1	0.441***	−0.031	−0.405***	−0.017
PM2.5						1	−0.095	−0.085	−0.139**
Temperature							1	−0.013	0.297***
Relative humidity								1	0.103*
Wind speed									1

* *p *≤ 0.05.

** *p *≤ 0.01.

*** *p *≤ 0.001.

Association between SBS symptoms and outdoor factors (see Table 5) was adjusted by personal factors (gender, current smoking and personal history of atopy) and meteorological parameters, depending on the type of exposure that was being assessed. Significant positive association was observed between outdoor exposure to nitrates (NO and NO2) and fatigue, with OR (95%CI) of 1.15 (1.04–1.29) and 1.14 (1.01–1.29), respectively. Outdoor SO2 was positively associated with cutaneous symptoms, mainly in scalp (1.25, 1.05–1.50) and hands (1.18, 1.00–1.39). Temperature was negatively associated with cutaneous symptoms in the scalp.

**Table 5:** Associations of exposure to outdoor air pollution, meteorological parameters, airborne pollens exposure and fungal spore exposure with SBS symptoms by multiple logistic regression model.

	General symptoms		Mucosal symptoms				Cutaneous symptoms		
	Fatigue	Headache	Ocular	Nasal	Oculo-nasal	Nasopharyngeal	Face	Scalp	Hands
	OR (95%CI)	OR (95%CI)	OR (95%CI)	OR (95%CI)	OR (95%CI)	OR (95%CI)	OR (95%CI)	OR (95%CI)	OR (95%CI)
Air pollution^a^									
NO	1.15 (1.04–1.29)**	1.02 (0.93–1.13)	0.94 (0.85–1.05)	0.98 (0.85–1.12)	0.94 (0.84–1.04)	0.89 (0.79–1.01)	0.96 (0.86–1.08)	0.97 (0.86–1.08)	1.04 (0.95–1.15)
NO2	1.14 (1.01–1.29)*	0.93 (0.82–1.05)	0.98 (0.86–1.11)	0.91 (0.76–1.07)	0.93 (0.81–1.06)	0.90 (0.78–1.05)	0.94 (0.81–1.08)	0.91 (0.78–1.05)	1.04 (0.92–1.16)
NOx	0.84 (0.76–0.93)**	0.97 (0.88–1.06)	1.02 (0.92–1.13)	1.00 (0.87–1.15)	1.02 (0.92–1.14)	1.11 (0.98–1.26)	1.04 (0.92–1.17)	1.00 (0.89–1.12)	0.97 (0.88–1.06)
SO2	1.18 (0.97–1.44)	1.12 (0.94–1.33)	1.10 (0.92–1.30)	1.18 (0.98–1.43)	1.14 (0.95–1.36)	1.03 (0.87–1.22)	1.08 (0.91–1.28)	1.25 (1.05–1.50)*	1.18 (1.00–1.39)*
PM10	0.96 (0.94–0.99)*	0.99 (0.97–1.02)	0.99 (0.96–1.01)	0.98 (0.96–1.00)	0.98 (0.96–1.00)	0.99 (0.97–1.01)	0.97 (0.95–1.00)*	0.96 (0.93–0.98)***	0.97 (0.95–0.99)*
PM2.5	0.98 (0.92–1.04)	1.00 (0.94–1.06)	1.01 (0.96–1.07)	1.02 (0.96–1.09)	1.03 (0.98–1.10)	1.03 (0.98–1.09)	1.00 (0.94–1.06)	1.04 (0.98–1.10)	1.00 (0.94–1.05)
Meteorological parameters^b^									
Temperature	0.99 (0.92–1.06)	0.95 (0.89–1.02)	0.96 (0.90–1.02)	0.94 (0.88–1.01)	0.95 (0.89–1.01)	1.00 (0.93–1.06)	1.03 (0.96–1.10)	0.93 (0.87–0.99)*	0.98 (0.92–1.04)
Humidity	0.98 (0.96–1.00)	0.99 (0.97–1.01)	0.99 (0.97–1.00)	0.98 (0.96–1.00)	0.99 (0.97–1.00)	1.00 (0.98–1.02)	1.00 (0.98–1.02)	0.98 (0.97–1.00)	0.99 (0.98–1.01)
Pressure (atm)	1.01 (0.97–1.05)	0.96 (0.92–1.00)	1.00 (0.97–1.04)	1.00 (0.96–1.04)	1.01 (0.97–1.04)	0.99 (0.96–1.03)	1.00 (0.96–1.03)	1.02 (0.98–1.05)	1.00 (0.97–1.04)
Wind speed	1.05 (0.92–1.09)	1.06 (0.98–1.15)	1.01 (0.93–1.09)	1.02 (0.94–1.10)	1.00 (0.93–1.08)	1.05 (0.98–1.13)	0.99 (0.92–1.07)	1.05 (0.98–1.14)	0.99 (0.92–1.07)
Pollen^b^									
*Quercus spp.* ^†^	1.12 (0.46–2.67)	1.15 (0.51–2.60)	2.80 (1.24–6.33)*	2.55 (1.01–6.42)*	3.02 (1.35–6.76)**	0.89 (0.43–1.83)	0.81 (0.37–1.74)	2.86 (1.32–6.19)**	1.12 (0.54–2.34)
*Fraxinus spp* ^†^	1.25 (0.64–2.42)	1.37 (0.74–2.56)	0.90 (0.49–1.64)	1.01 (0.54–1.89)	0.97 (0.53–1.80)	1.20 (0.68–2.11)	1.92 (1.05–3.50)*	1.26 (0.70–2.28)	1.04 (0.59–1.85)
Fungi^c^									
*Aspergillus spp* ^†^	2.28 (0.91–5.68)	1.03 (0.50–2.09)	2.22 (1.04–4.72)*	2.08(0.93–4.69)	2.25(1.07–4.70)*	0.94(0.48–2.05)	1.34(0.66–2.72)	2.95(1.20–5.41)**	1.36(0.68–2.69)

^a^ Adjusted for gender, current smoking, history of atopy, external temperature and windspeed for the last—months.

^b^ Adjusted for gender, current smoking and history of atopy.

^c^ Adjusted for gender, current smoking, history of atopy, relative humidity and exposure to *Penicillium's* spores.

^†^ Results for “high amounts”, taking the “non present” category as reference.

* *p* ≤ 0.05.

** *p* ≤ 0.01.

*** *p* ≤ 0.001.

Airborne pollens exposure was positively associated with mucosal symptoms in eyes and nose, whether separately or together (3.02, 1.35–6.76), and cutaneous symptoms in scalp (2.86, 1.32–6.19) for *Quercus spp*, and cutaneous symptoms in face (1.92, 1.05–3.50) for *Fraxinus spp*.

Outdoor fungal spore exposure was positively associated with oculo-nasal (2.25, 1.07–4.70) and cutaneous symptoms in scalp (2.95, 1.20–5.41) for *Aspergillus spp*.

## Discussion

Mexico is experiencing rapid urbanization that negatively impacts the environment, particularly indoor and outdoor air quality, and has detrimental effects on human health.

To understand the phenomenon of sick building syndrome, it is essential to know the concept of indoor air quality, which implies exposure to both internal and external pollutants and the overlap that exists between them (10), as well as aeroallergens that represent an important source that contributes to pollution by modulating indoor aeroallergen concentrations.

The main objective of this study was to evaluate the associations between outdoor pollutants and sick building syndrome symptoms in the home environment, also as a secondary objective we evaluated the association between moderate, high, and very high levels of pollen and spores with the presence or absence of symptoms of sick building syndrome.

Within the characteristics of our population, the majority belonged to the female gender and 35% had a history of atopy. When evaluating the symptoms, fatigue was the most prevalent symptom followed by nasal symptoms and headache. In the study by Mentese et al. fatigue, cold-like symptoms and difficulty concentrating were described as the most prevalent (11). Lu et al. found a higher prevalence of fatigue and headache (6), consistent with our findings.

To date, no evidence has been found that any specific factor can cause sick building syndrome; however, there are various factors that can contribute to its exacerbation (12). The present study found that the main factors associated with the appearance of the symptoms are the personal characteristics of the subject, such as a personal history of atopy and female gender. A history of atopy was associated with all symptoms except headache, and the female gender had a significant association with headache, fatigue, and skin symptoms, consistent with the findings of the study by Lu et al. (6). Kishi et al. found that having one or more allergic symptoms was significantly correlated with experiencing one or more SHS symptoms in both children and adults/adolescents (13). Our study found that women in the state of Nuevo León spend more time indoors, which increases exposure to polluting factors in this environment (14). This suggests a greater female gender risk of developing sick building syndrome.

Smoking is a known risk factor that negatively impacts human health. Znyk et al, carried out a systematic review on the adverse health effects of exposure to heated tobacco products, both *in vitro* and human investigations reveal that there may be a relationship between the use of tobacco products and the development of respiratory diseases, particularly due to its detrimental effects on lung physiology (15). In our study, 10.8% of the subjects reported positive smoking, predominantly in male gender. We did not find an increase in the presence of symptoms in the subjects who reported having active or passive smoking. One limitation when evaluating this variable was the small number of subjects who reported exposure to tobacco. Similar to our study Lu et al. (6), did not find significant correlations between the subject's smoking habit and their symptoms of SBS. On the other hand, in the study evaluating personal factors by Lu et al. they found that active smoking was associated with both upper respiratory symptoms (itchy nose, runny nose, nasal congestion, sneezing, and sore throat) as well as non-specific symptoms (headache, tiredness, difficulty, concentration, anger and dizziness) (16).

There is evidence from previous studies that evaluated and described an increase in the prevalence and incidence of sick building syndrome symptoms in relation to outdoor pollution and climatic factors (11). To evaluate these parameters, a multivariate logistic regression model was used, which included confounding factors that could affect the results, such as gender, a history of personal atopy, and active smoking, since the symptoms described in sick building syndrome are non-specific and are associated with respiratory and dermatological pathologies of allergic etiology or irritative factors such as tobacco smoke. Our results showed that there is an association between nitrogen dioxides and the presence of fatigue. In the study carried out by Zhang et al., a positive association of indoor and outdoor SO2 with the prevalence of symptoms was described; CO2, NO2 and relative humidity were positively associated with the recent onset of mucosal symptoms, headache, and fatigue and outdoor PM10 were positively correlated with newly developing skin, general, and mucosal symptoms (15). Lu et al. found a significant association between outdoor SO2 exposure and nasal symptoms (6). In our study, no significant associations were found with the rest of the pollutants analyzed that could serve as risk factors for the development of symptoms.

There are few studies evaluating the impact of spore levels in outdoor air on the symptoms of sick building syndrome. To the best of our knowledge this is the first study that compares pollen levels with the clinical manifestations of this syndrome. Our study evaluated the main pollen reported in the state of Nuevo León. A statistically significant association was found between elevated levels of *Quercus spp.* and the presence of ocular and nasal symptoms, as well as itching on the scalp and elevated levels of *Fraxinus spp.* pollen with skin symptoms on the face. In the Mentese et al. (11) study, factors that contribute to the alteration of indoor air quality are described, including the spread of outdoor-related mold through ventilation due to the contributions of mold levels and relative humidity. This study describes high levels of *Cladosporium spp.* considered as fungi from the outdoor environment that was considered as a potential factor for the alteration of indoor air quality.

In our study, very high values of *Cladosporium spp*, *Aspergillus spp and Penicillium spp* spores were found during the months of November to April. In addition, significant associations were found between elevated Aspergillus levels and the presence of oculo-nasal symptoms and itchy scalp.

According to the study carried out by Lu et al. (6), outdoor temperature was positively associated with ocular symptoms, while relative humidity was negatively associated with fatigue. In the present study we did not find significant associations between the variations of the meteorological parameters with the symptoms of the sick building syndrome.

One of the limitations of our study is that that we did not evaluate the indoor air quality (IAQ). Although statistical associations were made regarding outdoor pollutants and SBS syndrome due to outdoor quality levels, we cannot establish definite casuality. Another limitation considered is that other respiratory symptoms such as wheezing, cough or dyspnea were no included in the questionnaire.

## Conclusion

Despite the limitations of the study, we can conclude that there is an association between the clinical manifestations of sick building syndrome with indoor and outdoor pollution. This study found that the most frequent symptoms related to sick building syndrome are fatigue, nasal symptoms and headache in the population of Monterrey and the metropolitan area. Our study also found significant associations with female gender and a history of atopy, which suggests a higher risk for these subjects.

This set of symptoms can coexist with many diseases, especially with the spectrum of allergic diseases, which justifies its intentional search in patients with a torpid evolution despite adequate management.

Sick building syndrome is still not well recognized among all medical personnel, and it generally goes unnoticed at the time of evaluation, the symptoms that characterize it are nonspecific, and there are no diagnostic tools to confirm its presence.

There are few studies that evaluate its impact on human health, and we consider it relevant to deepen its knowledge since if it is identified and the corresponding control measures are carried out, it could reduce the morbidity of other associated diseases.

## Data Availability

The original contributions presented in the study are included in the article/**Supplementary Material**, further inquiries can be directed to the corresponding author.
