# Supplementary material for: Sick building syndrome: do outdoor pollutants and pollen affect it?
Source: Front Allergy. 2024 Jul 5;5:1383079. doi: 10.3389/falgy.2024.1383079 (PMC11257870; doi:10.3389/falgy.2024.1383079)
Supplement: Supplementary file 1 [file Datasheet1.pdf]

Supplementary form. Questionnaire applied to subjects

Contaminación intramuros, extramuros y su relación con las manifestaciones clínicas del síndrome del edificio enfermo

La siguiente encuesta forma parte de un estudio de investigación por lo que es muy importante que lea y comprenda la siguiente información. Este estudio tiene como propósito encontrar la relación de la contaminación ambiental tanto interna como externa con los síntomas del síndrome del edificio enfermo el cual es un conjunto de molestias y enfermedades ocasionadas por cuestiones físicas de los edificios y por algunas partículas contaminantes que se pueden encontrar dentro y fuera del hogar. De esta manera podemos contribuir al desarrollo de estrategias de prevención por parte de los habitantes del edificio.

Su participación en este estudio es estrictamente voluntaria y gratuita. En cualquier momento puede negarse a participar o completar la encuesta sin que su atención médica se vea afectada. Si usted acepta participar en este estudio nuestro equipo de investigación se encargará de recabar información sobre su salud y las características de su hogar. Esta información es estrictamente confidencial e incluye datos personales como su domicilio (colonia) y el municipio en el que habita, la información recabada en este estudio se guardará como base para poder desarrollar estudios futuros y puede ser presentada en reuniones académicas o en publicaciones científicas, siempre cuidando su confidencialidad.

La presente investigación está aprobada por el Comité de Ética en Investigación del Hospital Universitario "Dr. José Eleuterio González" y el equipo de investigación será el responsable de salvaguardar la información de acuerdo con las regulaciones locales, nacionales e internacionales. En caso de tener alguna pregunta relacionada a sus derechos como participante podrá contactar al Dr. José Gerardo Garza Leal, Presidente del Comité de Ética en el Hospital Universitario "Dr. José Eleuterio González". En caso de tener dudas en relación a sus derechos como participante podrá contactar al Lic. Antonio Zapata de la Riva. Comité de Ética en Investigación del Hospital Universitario "Dr. José Eleuterio González". Av. Francisco I. Madero y Av. Gonzalitos s/n, Col. Mitras Centro, Monterrey, Nuevo León, México. CP 64460. Teléfonos: (81) 83294000 ext 2870 a 2874, Correo electrónico: investigacionclinica@meduanl.com

**Titulo del estudio**	"Contaminación intramuros, extramuros y su asociación con las manifestaciones clínicas del síndrome del edificio enfermo"
**Nombre del Investigador Principal**	Dra. med. Sandra Nora González Díaz
**Servicio / Departamento**	Centro Regional de Alergia e Inmunología Clínica, Hospital Universitario "Dr. José Eleuterio González", Monterrey, N.L. México
**Teléfono de Contacto**	8183462515
**Persona de Contacto**	Dra. med. Sandra Nora González Díaz
**Versión de Documento**	Versión 1.0
**Fecha de Documento**	Julio de 2021

*

- ◯ Deseo participar y acepto los términos y condiciones
- ◯ No deseo participar

Siguiente   Borrar formulario

Cuestionario

Género: *

- ◯ Mujer
- ◯ Hombre

Edad: (ingrese únicamente el número) *

Tu respuesta

Municipio en el que habita: *

- ◯ Monterrey
- ◯ Apodaca
- ◯ Cadereyta Jiménez
- ◯ El Carmen
- ◯ García
- ◯ San Pedro Garza García
- ◯ General Escobedo
- ◯ Guadalupe
- ◯ Juárez
- ◯ Salinas Victoria
- ◯ San Nicolás de los Garza
- ◯ Santa Catarina
- ◯ Santiago
- ◯ Zuazua

¿Qué antigüedad tiene su casa? *

- ◯ Menos de 10 años
- ◯ Más de 10 años

Tipo de vivienda en la que habita: *

- ◯ Departamento (<75m2)
- ◯ Casa (>75m2)

Edificio Consulta Externa 2, 4º Piso
Av. Francisco I. Madero Pte. s/n y Av. Gonzalitos,
Col. Mitras Centro, Monterrey, N.L., México, C.P. 64460
Tel. (81) 8346-2515 8347-6798

Número de piso en el que habita *

- ○ 1
- ○ 2
- ○ 3
- ○ 4
- ○ >5

¿Cuántas horas al día pasa en casa? *

- ○ De 18 a 24 horas
- ○ De 12 a 18 horas
- ○ Menos de 12 horas

¿Cuántas personas viven en su hogar? (ingresar solamente el número) *

Tu respuesta ____________

Rango de edades de las personas que habitan en la vivienda (favor de marcar en el cuadro número de personas en ese rango de edades)

	1	2	3	4	>5
0 a 18 años	☐	☐	☐	☐	☐
19 a 40 años	☐	☐	☐	☐	☐
41 a 60 años	☐	☐	☐	☐	☐
Más de 61 años	☐	☐	☐	☐	☐

¿Cuánto años tiene habitando esta casa? (ingresar solamente el número de años) *

Tu respuesta ____________

¿Qué tipo de sistema de enfriamiento utiliza en su hogar? *

- ○ Ninguno
- ○ Aire acondicionado (AIRE LAVADO)
- ○ Aire centralizado
- ○ Minisplit

¿Cuántas habitaciones hay en su hogar (dormitorios, baños, cocina, sala, estancia, etc.)? (Ingrese únicamente el número) *

Tu respuesta

¿Qué tipo de estufa se utiliza en casa? *

- ○ Estufa de gas
- ○ Estufa de leña
- ○ Parrilla eléctrica

¿Qué combustible utiliza para cocinar? *

- ○ Gas butano - propano
- ○ Electricidad
- ○ Gas natural
- ○ Carbón
- ○ Madera

¿Qué tan seguido abre las ventanas de su casa? *

- ○ Nunca
- ○ A veces
- ○ Frecuentemente

¿Qué tipo de piso tiene en su casa? *

- ○ Madera
- ○ Concreto
- ○ Cubierto de alfombra
- ○ Cerámica (Azulejo)

Seleccione la opción que mas corresponda de acuerdo a su casa en relación a la humedad *

- ○ Condensación del cristal de las ventanas en invierno
- ○ Daños en las paredes o techo por agua
- ○ Humedad en la cama o ropa
- ○ Manchas de humedad en el piso, cancelería o techo
- ○ Olor a moho

¿Qué fuente de iluminación utiliza en su hogar? *

- ◯ Electricidad
- ◯ Lámparas de queroseno
- ◯ Velas

¿Qué mascota tiene en casa? *

- ☐ Ninguna.
- ☐ Gato
- ☐ Perro
- ☐ Aves
- ☐ Otra...

¿Tiene plantas dentro de casa? *

- ◯ Sí
- ◯ No

¿Alguien fuma dentro de casa? *

- ◯ Sí
- ◯ No

Edificio Consulta Externa 2, 4º Piso
Av. Francisco I. Madero Pte. s/n y Av. Gonzalitos,
Col. Mitras Centro, Monterrey, N.L., México, C.P. 64460
Tel. (81) 8346-2515 8347-6798

Factores de riesgo en el área de trabajo

Sólo contestar en caso de ser trabajador activo de lo contrario proceder a siguiente sección

¿Cuántas horas al día pasa en su lugar de trabajo? (Ingrese únicamente el número)

Texto de respuesta corta

¿Qué tipo de sistema de climatización se utiliza en su lugar de trabajo?

- ◯ Ninguno
- ◯ Aire acondicionado (aire lavado)
- ◯ Aire centralizado
- ◯ Minisplit

¿Qué tipo de piso tiene su lugar de trabajo?

- ◯ Madera
- ◯ Concreto
- ◯ Cubierta de alfombra
- ◯ Cerámica (Azulejo)

¿Considera su lugar de trabajo un ambiente libre de humo de tabaco?

- ◯ Sí
- ◯ No

Después de la sección 3 Ir a la siguiente sección

Antecedentes personales

Continuación

¿Usted fuma actualmente? *

- ○ Sí
- ○ No

¿Ha sido diagnosticado por un médico con alguna de las siguientes enfermedades? *

- ☐ Rinitis alérgica/Sinusitis
- ☐ Conjuntivitis alérgica
- ☐ Dermatitis atópica
- ☐ Asma alérgica

¿Se ha sentido fatigado (cansado) en los últimos 3 meses? *

- ○ Sí, a menudo
- ○ Sí, a veces
- ○ No, nunca

¿Ha tenido dolor de cabeza en los últimos 3 meses? *

- ○ Sí, a menudo
- ○ Sí, a veces
- ○ No, nunca

¿Ha tenido comezón, ardor o irritación de los ojos en los últimos 3 meses? *

- ○ Sí, a menudo
- ○ Sí, a veces
- ○ No, nunca

¿Ha tenido escurrimiento nasal (moco transparente), congestión o irritación de la nariz en los últimos 3 meses? *

- ○ Sí, a menudo
- ○ Sí, a veces
- ○ No, nunca

¿Ha sentido la garganta seca o ha tenido la voz ronca en los últimos 3 meses? *

- ○ Sí, a menudo
- ○ Sí, a veces
- ○ No, nunca

¿Ha notado la piel de su cara seca o enrojecida en los últimos 3 meses? *

- Sí, a menudo
- Sí, a veces
- No, nunca

¿Ha notado descamación o picazón en el cuero cabelludo en los últimos 3 meses? *

- Sí, a menudo
- Sí, a veces
- No, nunca

¿Ha sentido sequedad, picazón o enrojecimiento de las manos en los últimos 3 meses? *

- Sí, a menudo
- Sí, a veces
- No, nunca

¿Estos sintomas son más frecuentes en su casa o en su trabajo? *

- Casa
- Trabajo

Edificio Consulta Externa 2, 4º Piso
Av. Francisco I. Madero Pte. s/n y Av. Gonzalitos,
Col. Mitras Centro, Monterrey, N.L., México, C.P. 64460
Tel. (81) 8346-2515 8347-6798
